# Disability Grant: a precarious lifeline for HIV/AIDS patients in South Africa

**DOI:** 10.1186/s12913-015-0870-8

**Published:** 2015-06-09

**Authors:** Veloshnee Govender, Jana Fried, Stephen Birch, Natsayi Chimbindi, Susan Cleary

**Affiliations:** Department of Public Health and Family Medicine, Health Economics Unit, University of Cape Town, Cape Town, South Africa; Centre for Agroecology, Water and Resilience, Coventry University, Coventry, 829 UK; Centre for Health Economics and Policy Analysis, McMaster University, Hamilton, Canada; Africa Centre for Health and Population studies, University of KwaZulu-Natal, Durban, South Africa

**Keywords:** HIV/AIDS, Healthcare access, Disability grant, South Africa

## Abstract

**Background:**

In South Africa, HIV/AIDS remains a major public health problem. In a context of chronic unemployment and deepening poverty, social assistance through a Disability Grant (DG) is extended to adults with HIV/AIDS who are unable to work because of a mental or physical disability. Using a mixed methods approach, we consider 1) inequalities in access to the DG for patients on ART and 2) implications of DG access for on-going access to healthcare.

**Methods:**

Data were collected in exit interviews with 1200 ART patients in two rural and two urban health sub-districts in four different South African provinces. Additionally, 17 and 18 in-depth interviews were completed with patients on ART treatment and ART providers, respectively, in three of the four sites included in the quantitative phase.

**Results:**

Grant recipients were comparatively worse off than non-recipients in terms of employment (9.1 % vs. 29.9 %) and wealth (58.3 % in the poorest half vs. 45.8 %). After controlling for socioeconomic and demographic factors, site, treatment duration, adherence and concomitant TB treatment, the regression analyses showed that the employed were significantly less likely to receive the DG than the unemployed (p < 0.001). Also, patients who were longer on treatment and receiving concomitant treatment (i.e., ART and tuberculosis care) were more likely to receive the DG (significant at the 5 % level). The qualitative analyses indicated that the DG alleviated the burden of healthcare related costs for ART patients. Both patients and healthcare providers spoke of the complexity of the grants process and eligibility criteria as a barrier to accessing the grant. This impacted adversely on patient-provider relationships.

**Conclusions:**

These findings highlight the appropriateness of the DG for people living with HIV/AIDS. However, improved collaboration between the Departments of Social Development and Health is essential for preparing healthcare providers who are at the interface between social security and potential recipients.

**Electronic supplementary material:**

The online version of this article (doi:10.1186/s12913-015-0870-8) contains supplementary material, which is available to authorized users.

## Background

*“Everyone has the right to have access to health care services (…) and social security, including, if they are unable to support themselves and their dependants, appropriate social assistance.” (Section 27 of the Bill of Rights, South Africa)* [1]

In South Africa (SA), HIV/AIDS remains a major public health problem. In 2010, approximately 5.5 million were infected with HIV, making it globally the country with the largest number of people living with the disease [2]. In a context of chronic unemployment and deepening poverty, two important government initiatives have the potential to ameliorate the additional socio-economic burden and costs of healthcare arising from HIV/AIDS for affected individuals. First, the provision of free primary health care has improved access to healthcare. In tackling the HIV epidemic, approximately 1.4 million people or three quarters of the eligible adults received free antiretroviral treatment (ART) by 2012 [2], making it the largest public ART programme in the world. Second, the existing social security system has been significantly expanded to reach a larger part of the population, specifically aimed at redressing past inequities arising from the apartheid system [3]. Here, the constitutional right to social security is of great relevance, with social security being “an important safety net that helps relieve poverty and protects people against economic shocks” [4]. Social assistance is also extended to adults with HIV/AIDS who are unable to work because of a mental or physical disability^1^ and are deemed eligible to receive a Disability Grant (DG).^2^ Approximately 16.0 million social grant payments were made to vulnerable people in January 2013, of which approximately 1.2 million were paid out as DGs [5].

Eligibility to the DG is defined by the South African Social Security Agency’s (SASSA’s) guidelines [6]. Criteria for qualification include applicant’s status (i.e., citizen, permanent resident or refugee); being of working age (18 years and older); passing a means test and having received a medical assessment report. The disability is assessed and confirmed by a medical assessor, who is a state appointed doctor. The DG can be either permanent (for disabilities lasting longer than 12 months) or temporary (for disabilities between six and 12 months) and HIV patients are eligible for the temporary DG.^3^ The DG is re-assessed and the person is re-examined by a doctor every six-months to confirm whether they still qualify. Hence, ART patients lose their DG as soon as their health begins to improve.^4^

It has been argued that eligibility to the DG and in particular the relative vagueness of definitions of ‘people with disability’ for people with HIV/AIDS has provided a gap for subjective interpretations for both applicants and the healthcare providers including doctors appointed as medical assessors [7, 8]. Although there are some key principles that should guide disability assessments, previous research indicates that the medical assessment report which is necessary to confirm disability is complex, particularly in relation to eligibility for the temporary and permanent DG [9–12]. Moreover, the on-going challenge of human resource capacity in the public health care sector, particularly the shortage of doctors, means that few staff are available to assess disability in public facilities, leaving very little time (as little as 3 min) to assess a patient’s disability [8].

McIntyre et al. [13] present a conceptual framework in which access to care is represented by the degree of fit between the characteristics and contexts of individuals with health care needs and the way the service is provided. The framework identifies three separate dimensions of access; affordability (do the individuals have the capacity to incur the full costs of receiving care?), availability (is the appropriate care supplied?) and acceptability (is care supplied in a way that meets the reasonable expectations of patients?). Both Cleary et al. [14] and Fried et al. [15] argue that although ART treatment is provided free of cost at facilities in the public sector, some patients are still facing financial challenges in accessing care.

Building on the findings of Cleary et al. [14] and informed by the access-framework of McIntyre et al. [13], this paper assesses predictors of access to the DG and how receiving a DG improves affordability to transport and food which may in turn impact on other access barriers such as availability and acceptability of services. We aim to investigate the interaction between access to the DG and access to care for HIV/AIDS. More specifically, we are guided by the following two research questions. Are there any inequalities in access to the DG for patients on ART? What are the implications of DG access for on-going access to healthcare? In relation to the access-framework, variations in ability to meet the shadow price of health care (i.e., the non-treatments costs associated with access to care) are expected to be associated with variations in service utilisation. DGs are considered a means of empowering those with limited means to meet the costs of accessing care.

We use data from patient-exit interviews across two urban and two rural sites in SA to investigate presence of inequalities in access to the DG. We were interested in the socio-economic, physical location and treatment characteristics of DG recipients (compared to non-grant recipients). Relevant is also the healthcare delivery system in which the patients’ access to care has an influence on DG uptake; some facilities may be structured to promote, encourage and provide support and information to patients regarding their social rights including application for DGs. In addition, some sites are more urban than others and the composition of socio-economic backgrounds of patients is likely to vary by site.

We explore underlying factors which either facilitate or inhibit access to the DG and how these differences in access play out in terms of healthcare access through patients’ narratives (both grant and non-grant recipients) and healthcare providers’ narratives. Uptake of the DG might be determined by patients’ knowledge and perceptions about the existence, eligibility conditions and the DG application process. Previous research indicates that access to information and knowledge about where to access care or services empowers an individual [13, 15–17]. We also consider healthcare providers’ knowledge about DGs and their ability to assist patients in accessing DGs and the implications for the acceptability of care.

## Methods

The study design was sequential mixed methods, using both quantitative and qualitative data collection techniques. Methodologically, this approach provides the opportunity to triangulate objective, quantifiable data with subjective, experiential data. The quantitative study (QUAN) employed patient exit interviews using a self-administered structured questionnaire, followed by fewer, but detailed qualitative in-depth interviews (qual) with patients and providers (i.e., Morse’s QUAN-qual taxonomy) [18]. The data were integrated in analysis for complementarity, which seeks “elaboration, enhancement, illustration, clarification of the results from one method with the results from the other method” ([19], p259).

### Quantitative methods

#### Sampling

Four health sub-districts in different provinces were selected as sites for this research, two in urban areas (Mitchells Plain in the Western Cape province and Soweto in Gauteng province) and two in rural areas (Bushbuckridge in Limpopo province and Hlabisa in KwaZulu-Natal province). The rural–urban selection was designed to capture different geographic locations; while four different provinces were selected to allow for insights from different governance contexts (this is particularly important given the ‘federal’ structure of South Africa, where the provinces have considerable decision-making autonomy). Key officials in the national and provincial health departments were also consulted in finalising the selection of sub-districts. User partners (i.e., senior managers responsible for the services included in the study) contributed to the final selection of study sites by identifying sub-districts classified as priority areas for utilisation and access evaluation. Finally, geographic access of the investigators to the sub-districts was also considered.

A two-stage sampling approach was used in each sub-district, first selecting a representative sample of health facilities, then within these facilities, a representative sample of users. For ART, all accredited facilities were included where possible, and where multiple facilities existed, self-weighting stratified, proportional or probability proportional to size methods were used to select facilities, again using routine data on the total number of users in each facility at the time of the research. Within each chosen facility, a random sample of patients was interviewed until the proposed facility sample size was reached. In total, a minimum of 300 patients were interviewed per sub-district; the planned sample size was therefore 1200 respondents.

#### Data collection and capture

Patient exit interview questionnaires were developed to collect demographic and socio-economic data as well as information on access to DGs and aspects of access to health care (Additional file 1). A range of measures of socio-economic and demographic status were collected, including employment status, total household consumption expenditure, whether or not the household received social grants and the value of those grants, and a composite asset index. The questionnaire was administered by trained interviewers in the language of the respondent’s choice. Completed questionnaires were checked for accuracy by a data collection coordinator and double entered into a data entry platform specifically designed for this purpose in Epidata.

#### Data analysis

Data were analysed using Stata/IC 11.0. Data were described using summary statistics, and logistic regressions were run to determine the characteristics of respondents receiving DGs. A composite asset index was created using multiple correspondence analysis (MCA) on several household level variables including type of house, material of walls, type of toilet, primary source of energy for cooking and ownership of assets such as a vehicle, fridge and livestock etc. Given the different governance contexts and different levels of non-governmental organisation (NGO) involvement between communities, we included dummy variables for the study sites to explore possible site effects. We also included variables relating to patient self-reported adherence (ever missed a visit, ever missed medication) and duration on treatment. We included concomitant treatment as an explanatory variable in the analysis. Since the presence of HIV/AIDS increases the risk of TB, there may be contamination within a particular patient population. For example, among individuals with TB, those receiving concomitant treatment for HIV/AIDS may be more likely to be receiving the DG because of the support and counselling provided through ART services. Demographic and socioeconomic variables were entered in the equations as control variables. The average exchange rate over the period of data collection was 1 US dollar to 9.03 South African Rands.

### Qualitative methods

#### Sampling

In-depth interviews were conducted with seventeen ART patients and eighteen ART providers purposively selected in three of the four sites included in the quantitative phase. The rural site Hlabisa was excluded from the qualitative phase due to project-related resource constraints.

Purposive sampling guided the selection of study participants. The selection criteria for patients included capturing a range of patient treatment experiences, indicated by patients being classified as either successful and unsuccessful (i.e., with treatment interruptions or treatment failure); gender; and groups identified through literature and preliminary data analysis in the quantitative phase as most likely to be treated differently by providers based on several factors (e.g., age, nationality, ethnicity, socio-economic status). To be able to capture the experiences of both successful and unsuccessful patients required recruiting interviewees beyond the treatment facilities through other routes including non-governmental organisations and patient networks. The selection criteria for providers included staff at different levels of seniority within the facility, different cadres of staff (professional, administrative and community treatment supporters) and length of service. Interviews were continued until the selection criteria were met and ‘saturation’ occurred, i.e., until no more additional themes describing access to care for patients on ART emerged. Access to DGs was one of these important themes.

#### Data collection and capture

Semi-structured interview guides were developed for patients (Additional file 2) and providers. Interviews with patients explored patient’s life histories (i.e., social support systems, education, income, migration, work), illness trajectories (i.e., from illness onset to diagnosis and treatment, treatment seeking, stigma) and experiences with the health care system (i.e., barriers constraining access and engagements with health care providers). The life and illness histories, social support narratives and experiences with the health care system were related as narratives which linked, as the patient saw it, the role of ART in everyday life [20]. Follow-up interviews were conducted with patients to clarify uncertainty over timeline and care-seeking events, where necessary. This also provided an opportunity to explore access issues in greater depth. As a caveat, we did not set out to investigate the role of DGs specifically at the beginning of this study; however, these emerged as an important issue in patients’ narratives that is worthy of further analysis.

Interviews with providers covered a range of topics including education and training, career trajectories, family histories, relationships with patients, colleagues and supervisors. Interviews also allowed for the exploration of constraints and challenges providers faced in fulfilling their roles and their perceptions of the access barriers faced by patients. Here, complexities around DGs and access to grants were described.

#### Data analysis

The transcripts were thematically coded in ATLAS ti.6 by two members of the research team. Coding was done both inductively and deductively. It was inductive in that the two researchers worked independently in reading and re-reading the transcripts and identified an initial set of codes, from which emerged several major themes such as patient and provider expectations. At the same time, the access-framework provided a conceptual lens from which hypotheses were derived (e.g., access to the DG alleviates transport costs and improves affordability, thereby improving adherence) and these hypotheses were tested against the data. The researchers then compared the similarities and divergences between their codes and themes to ensure reliability. To ensure rigour, codes and themes were shared with the wider research team and suggested modifications were discussed until consensus was reached. Broader themes which were derived both inductively and deductively were then related back to the theoretical framework. Quotations were selected based on their representativeness of the emerging themes.

### Ethical issues

The Universities of Cape Town, Witwatersrand and Kwa-Zulu Natal and the South African Provincial Health Research Committees granted ethical clearance. Informed, written consent to participate in the study was obtained from each participant. Consent was also obtained to publish the patient data, subject to the names being anonymised. Participants were only interviewed if they were over 18 years of age. In order to ensure patient and provider confidentiality, interviews were anonymised and quotations are presented using pseudonyms and regional location only.

## Results

### Characteristics of recipients of a Disability Grant

Table 1 summarises the demographic, socio-economic and treatment characteristics of ART patients in our sample according to whether they were recipients of a DG or not. Approximately a third (36.5 %) of all ART patients (*n* = 1267) received the DG across the four study sites. Unemployment levels were high among both grant recipients (90.9 %) and non-recipients (70.1 %). There was no significant difference in the demographic characteristics (i.e., age-sex composition) between grant and non-grant recipients. Significant differences were found with respect to socio-economic and treatment characteristics between the two groups. Grant recipients were comparatively worse off than non-recipients in terms of socio-economic indicators of employment (9.1 % vs. 29.9 %) and wealth, measured by the asset index (58.3 % in the poorest half vs. 45.8 %). With respect to treatment variables, grant recipients reported longer treatment duration than non-recipients. Also, a relatively smaller number of grant recipients compared to non-recipients reported poorer treatment adherence (i.e., missed clinic visits and missed medication dosages).

**Table 1:** Characteristics of the recipients of disability grants and determinants of receipt

Variables	Category/measure	(*n* = 1267)	ART	AOR	p-value
		In receipt of a grant	Not in receipt of a grant		
		(*n* = 463)	(*n* = 804)		
Age	Median	39.00	35.00	1.01	0.14
Sex	Male	29.37	24.53	1.22	0.19
	Female	70.63	75.47	Referent	
Employment	Employed	9.13	29.89	0.23	0.00
	Unemployed	90.87	70.11	Referent	
Asset index	Wealthier	41.68	54.17	1.10	0.57
	Poorer	58.32	45.83	Referent	
Education	None or some basic	42.64	30.14	Referent	
	Some secondary	43.29	46.45	0.80	0.18
	Completed secondary	14.07	23.41	0.58	0.02
Household expenditure	Median	972.22	973.95	1.00	0.05
Site	Bushbuckridge	30.67	21.14	Referent	
	Soweto	16.85	31.47	0.37	0.00
	Hlabisa	30.89	19.53	0.96	0.81
	Mitchells Plain	21.60	27.86	0.57	0.01
Duration on treatment	Median	12.00	10.00	1.01	0.02
Reported missing clinic visit(s)	Yes (%)	4.54	5.86	1.10	0.75
Reported missing medication dosage(s)	Yes (%)	9.72	16.10	0.81	0.33
Receiving concomitant TB treatment or ART	Yes (%)	11.45	9.10	1.68	0.02

### Predictors of access to the Disability Grant

Table 1 also indicates the adjusted odds ratios (AOR) of receiving the DG for each demographic, socio-economic and treatment variable. The data suggest that there was no relationship (i.e., AOR is either close to or is 1) between grant receipt and age, sex, asset index, education, and treatment adherence variables. Unemployment was significantly associated with receipt of the DG (at the 1 % level). The employed were significantly less likely to receive the grant than the unemployed. Significant (at the 5 % level) treatment variables were treatment duration and those patients receiving concomitant treatment for ART or TB (i.e., being treated for both conditions). We also saw a relationship by site. Patients in the two urban sites (i.e., Soweto and Mitchells Plain) were significantly less likely to receive a grant than patients in the two rural sites (i.e., Bushbuckridge and Hlabisa). However the likelihood of receiving a grant was not significantly different between patients in the two rural sites.

In order to understand the role of the DG in enabling treatment access and sustaining livelihoods and to illuminate our quantitative findings further, we analysed the patient and provider narratives.

### Role of Disability Grant in enabling the uptake of treatment

In a context of chronic poverty and high unemployment, conversations with both patients and providers converged around the centrality of DGs as a lifeline to survival. Some of the excerpts presented are indicative of the many ways in which DGs contribute to ameliorating patients’ everyday challenges that are confounded by the need to access and adhere to treatment:

*It helps me to buy food in the house and take my child to school. (Patient Tshidiso Mlahleki, Bushbuckridge)*

Providers were empathetic about the often desperate circumstances of patients and understood well their dependency on the DG:

*The majority of our patients come from the township areas, so we talking sub-economic, we talking like the basic of the basics…some of them would complain they don’t have food, so a lot of them are on DGs (Provider Tasneem Essop, Mitchells Plain)*

The role of the DG in enabling patients to pay for transport fees to the facilities was a recurrent theme across patients’ and providers’ narratives.

*I’ve spoken to a lot of my patients…they would not come for appointments because of money…not having taxi fare (Provider Michelle May, Mitchells Plain)*

In the following quote, the patient referred to her inability to afford the transport costs to the facility once the DG stopped:

*I asked the money from my son, I asked him to give me R20.00, it is R10.00 a taxi from where I stay to come here* [to the health facility]. *… Even this R20.00, I don’t even know how I am going to pay it back, I don’t know. I’m not even able to work because I don’t have the strength.* (She started crying) *(Patient Silindile Zama, Bushbuckridge)*

In contrast to the above narratives which speak to the challenges of household survival and being able to access and follow treatment without the DG, patients receiving the DG described eloquently the ways in which it enabled access to treatment:

*I am feeling fine and even my wife she is feeling well… We are following all the instructions after receiving the grant. We buy everything that we’re supposed to eat and we also take our treatment accordingly. And I see a very big change in our life because we are feeling well and fine… (Patient Glen Mnsinsi, Bushbuckridge)*

As noted earlier, once an ART patient’s health improves and their CD4 count rises, they are considered able to work and they no longer qualify for the DG. In the absence of the DG, the survival of patients and their households was tenuous including treatment access:

*They cut it* [DG] *because they say I’m no longer sick, so now I have to live by borrowing money from people and paying it back is a challenge because when I do get that bit of money, the only thing I think about is to buy food for my kids…(Patient Silindile Zama, Bushbuckridge)*

### Administrative process for accessing the Disability Grant

Patients and providers also spoke of the administrative challenges experienced in obtaining the DG. Poor knowledge and often misconceptions of the grant process and the eligibility criteria were a recurrent theme in both patient and provider narratives, as evident in the following excerpt:

*…they do the height and weight, maybe they see the weight, and “No, this one can get the grant”, “This one doesn’t qualify because this one is working, this one is not working”. The others, their weight is right, so the doctor says to them “your weight is right, you can go and find a job, we can’t give you the grant”. (Provider Thembisile Shaka, Mitchells Plain)*

Poor provider knowledge of the DG process and eligibility criteria had implications for how patients understand the process, leading to confusion and mistrust:

*Yes, until today, they were refusing saying that they are looking for a patients whom is unable to do anything…They are looking for patient who is on the pushcart…You cannot get any [grant if you are able to walk]…They do not allow you to see the doctor. I do not know which procedure are they using to see a doctor; or maybe the patients are bribing the nurses to see the doctor, because when I want to see a doctor, they would not allow me and I would wait until 5 o’clock in the evening and until they knock off, still queuing to see a doctor. He just came for couple of hours then he left us behind…hey no, they* [healthcare providers] *were undermining me very much.. (Patient Mvelo Moyo, Bushbuckridge)*

In contrast, other patients were effectively supported and guided through the grant process and received their DG without unnecessary delay:

*Oh, to get grant I was told here at the clinic where I was attending HIV classes…I went to the hall (civic/community hall) where you get application forms…It was filled the way it was supposed to be filled and I brought it there when the time was right and it took two weeks. (Patient Ayabong Dlomo, Mitchells Plain)*

The government has attempted to stem the confusion regarding the eligibility criteria through workshops:

*The department tries…once in a year, we get workshops from them in terms of the criteria that they are using…so we try and come back and then implement those changes and then tell our patients that what is now looked at…you know like the CD4 count thing, they no longer use it. …They are saying it’s deceiving; they no longer use it because a patient can have a low CD4 count but they can still be well and able to work… (Provider Nonkululeko Ntuli, Soweto)*

Evident from the foregoing discussion, poor communication between patients and providers in relation to the application process and eligibility criteria for the DG had implications for the nature of the provider-patient relationship more generally. In some instances patients viewed providers as cooperative and facilitating access to the DG and in others providers were viewed with suspicion and as gatekeepers obstructing access to the DG. Often however, patients perceived providers as being unhelpful and even discriminatory:

*A grant like the people told me about…they say I am supposed to get one. But the people don’t say anything; they rather told me I stayed away too many days. I can’t help if I stay away for many days because I can’t make it every day. (Patient Mark Kriel, Mitchells Plain)*

This suggests that many patients perceived providers as unhelpful and even impeding their access to the DG. However, there was also suspicion among some providers, which influenced their perception and interaction with patients, often adversely:

*Then we get those who want the DG and they feel that if they finish the treatment, the DG is going to end! So, the next way to extend the DG is to default. It is not a lot of those, but you get these serial defaulters, you know they start getting better, and then they just disappear.* (Provider *Dr. Menzi Khumalo, Mitchells Plain)*

This contributed to a general sense of helplessness and disillusionment, often leaving them feeling discouraged and disempowered, as noted by patients:

*When I was applying for a DG, they never wrote full information on the application form. On top of that, when you go to see a doctor and tell him that he did not fill the forms in a right way, they do not want to take your story. For me it meant that I lost the grant, it has failed. (Patient Malusi Moloi, Soweto)*

In summary, the knowledge of the administrative processes used to determine eligibility and gain approval and the administrative process itself appeared to be unnecessarily onerous from the perspective of patients. Besides being disempowering, patients also suggested that alternative informal criteria were being used by those involved in the approval process. This was perceived as unfair and a barrier to accessing the DG which in turn was perceived as a barrier to accessing ART. At the same time, healthcare providers themselves expressed uncertainty about the eligibility conditions and process of applying for the DG.

## Discussion

This paper focuses on factors that explain variations in access to the DG, the administrative challenges patients experience in obtaining the grant and finally the role of the grant in improving treatment access and sustaining livelihoods of rural and urban ART patients in SA.

With regard to the first research question (i.e., are there inequalities in access to the DG for patients on ART?), the findings do not indicate systematic discrimination between patients based on socio-economic status or other demographic variables (i.e., age or sex). However, the quantitative analyses point to low overall coverage across the four sites which raises questions of whether there is a problem of targeting and uptake. According to the SASSA guidelines for 2007–08,^5^ households with annual income of less than R58,224 (or R4,852 per month) were eligible for the DG. In the sampled households, almost all households would be eligible for the DG, given an average household expenditure^6^ of approximately R972.00 per month. At the same time, while household income is a necessary criterion for the DG, the main criteria is proof of disability, which requires assessment by a clinician appointed for this task.

Importantly, employment was a significant predictor of access to the DG. Therefore, although the threshold level of income above which an individual did not qualify for the DG was relatively high, the probability of a patient failing to satisfy the means test was expected to be higher among employed patients than non-employed patients. In other words, the probability of employed patients receiving the DG was significantly lower than for unemployed patients. This might also suggest that the employed found it more difficult to attend the various stages of the assessment process because of difficulties in arranging time off work. Alternatively, it might suggest either appropriate targeting where employment is being used as an informal assessment either of means or of disability. With respect to the latter, estimating or verifying a patient’s means may be difficult and time consuming and so alternative ‘markers’ or proxies (e.g., employment status) may be used by assessors in order to expedite the process (irrespective of the actual per capita income of the household). However, whether this was a practice in all or any of the sites cannot be verified and established from our analysis. But it does imply that more attention needs to be given to the assessment process.

There are observed inequalities in access across sites, with higher levels of DG coverage in rural sites. Patients in the two urban sites are significantly less likely to receive a grant than patients in Bushbuckridge. A possible explanation might be that rural patients are more likely to be unemployed and poorer and therefore more likely to qualify for grants than those in urban sites. It is also possible that socio-cultural (e.g., stigma) factors could play a part in contributing to these observed differences between the rural and urban sites. Furthermore, in each sub-district setting, we would expect different implementation practices given the discretion that implementing actors have regarding service design, including location, opening hours and referral mechanisms, as well as the local practices of the doctors that have been appointed as medical assessors. Finally, our provider interviews indicate that at the facility level, there are differences in the ways in which specific ART providers communicate with patients about the grant, and the extent to which they might go the extra mile to facilitate access for their patients (see also Elloker et al. [21]).

The interviews with providers and patients point to the complexity of the DG as a challenge for access. As Black Sash, an advocacy group has argued “…there has been no agreed definition of disability or consistent application of a standardised tool to assess disability. This has subjected many of our clients to the discretion of medical practitioners and officials, which contradicts the basic principles of administrative justice.” ([22], p3).

Complexity of the grants process and eligibility contributed to delays in access to the grant. As Knight et al. ([12], p143-144) found in their qualitative study of households in rural KwaZulu-Natal “… the relative timing of receipt of the disability grant and beginning ART is important. In contrast to those on ART who received the grant in good time, those who received it late recovered health more slowly… suggest [ing] that a synergistic relationship may exist between timeous receipt of this grant and improved health outcomes on ART.”

The imperfect communication and implementation practices around the DG and the subsequent misunderstandings of its processing have impacted relationships and communication between patients and healthcare providers. This contributed to suspicion and mistrust over the motives of the other. Providers spoke of patients attempting to defraud the system. The perception of providers needs to be examined against the fact that ART patients often show a reduction in symptoms and ‘disability’. Healthcare providers argued that patients, who are dependent on the DG, tried to avoid a situation where they were no longer considered disabled once their health improved. In the absence of employment opportunities or alternative social assistance mechanisms, this, as Simchowitz ([23], p12) points out, can create a potentially “vicious cycle of sickness and health,” where discontinuation of the DG might contribute to the health of patients deteriorating substantially enough to again qualify for a DG. Similarly, Natrass ([24], p14) speaks of “perverse incentives”, where patients are left to choose between treatment and health or stronger economic security for themselves and their family. On the other hand, patients speak of being ‘denied’ the grant and of fraudulent and discriminatory practices on the part of the providers. A recent study exploring the perceptions and experiences regarding the DG for persons on ART in South Africa concluded that “Participants valued their health more than the income, however, and, despite the risk of losing the grant, remained adherent to ART” [25]

Importantly, patients might view healthcare providers as fulfilling a role which extends to being gatekeepers of information and either facilitators or barriers to accessing the DG. One might further expect that the nature of the patient-provider relationship around the DG has implications for and spills over onto the ART relationship. As previous research has shown, the social relationship between patients and providers is crucial for treatment adherence and success [26]. Further, Noyes and Popay ([27], p238) argue “misunderstanding and miscommunication between healthcare professionals and services users appear to be commonplace.”

This paper provides additional evidence of the role of the DG in sustaining the livelihoods of often impoverished ART patients and also enabling treatment access. This is consistent with the finding that household expenditure was similar for both grant-recipients and non-recipients, suggesting that grant recipients could afford higher expenditure levels owing to the grant. Therefore, one might presume that the grant ‘fills the gap’ and enables those who receive it to achieve the same levels of household expenditure as the non-recipients. These findings are consistent with the findings of previous research on the role of the DG in the context of HIV/AIDS [28]. Conversely, withdrawal of the DG, once the health status of the grantee improved impacted adversely on the lives of patients and their ability to adhere to treatment (ibid). Even with access to the DG, patients might employ a variety of other factors to cope with health care costs, including borrowing money, selling assets and receiving support from family. Fried et al. [15] use ART patients’ narratives to highlight the challenges of accessing ART over a long period of time and emphasise the importance of social support networks. A related paper [14] provides futher details of these alternative support methods, and contrasts the experience of ART patients to the experiences of individuals using TB and maternity services.

In recent years, there have been increased calls for the introduction of a Chronic Illness Grant [8, 12, 22, 29, 30]. As argued by Black Sash ([22], p4), “…we do not think it is appropriate for people who have chronic illnesses to necessarily be defined as disabled, as it both misrepresents these people’s potential for health and works perversely against health-affirming behaviours.” The Chronic Illness Grant would serve to provide life-long income support for those with chronic illness including HIV/AIDs and unlike the DG would not be discontinued once the patient’s health has improved and the disability has gone. It would also not prejudice those patients who are employed.

This study has several limitations. Firstly, we did not have data on patients’ disability. This information would have allowed us to be able to comment further on patients’ eligibility for the DG. Secondly, at the time of our study, we did not interview a state-appointed doctor who assesses patients’ eligibility for the DG. This, given substantial variations in policy implementation between different provinces, would have called for a substantial expansion of our data collection strategy. Thirdly, the rural site Hlabisa was excluded from the qualitative component of the research. Therefore, we are unable to identify reasons why patients in Hlabisa had better access to the DG than those in the other sites. Fourthly, the cross-sectional design of the quantitative component does not allow us to comment on the relationship between access to the DG and treatment adherence. More specifically, we cannot provide evidence based on the quantitative analysis that DG recipients are more likely to be adherent (i.e., successful use) than non-recipients. The patients’ exit interviews do not really explain or illuminate successful use very clearly since few people reported low adherence or missing visits. Further, non-adherent patients were less likely to be captured in the study, which is a research design issue. Finally, for the qualitative component, recruitment and follow-up of patients was challenging. In some instances, although appointments were arranged and interviewees were reminded about the date of the interview, they did not meet the appointment. Often cited reasons included conflict between appointment and household or work responsibilities and lack of transport money to the clinic. Also, tracing patients was complicated by the fact that many of them did not have a permanent address, or had provided an incorrect address or were not contactable by telephone.

## Conclusion

A key finding of this paper is that in a context of chronic unemployment and high levels of poverty, employment is an important predictor of access to the DG. In addition, there does not appear to be systematic discrimination between patients based on socio-economic status, age or gender. Further, access to the DG is a life-line for ART patients. It enables them to meet either fully or partially the healthcare related costs including transport, food and other household costs and enables treatment access. In the case of HIV/AIDS where regular visits to health care facilities and regular food intake are required, the limited capacity to borrow money or sell assets may represent a serious constraint on an individual’s ability to adhere to recommended treatment plans. Where the treatment is for infectious conditions–and particularly for those that carry a high risk of mutation and drug resistance–there is a clear public interest to provide patients with every opportunity to access and adhere to treatment. As argued in South Africa’s Bill of Rights:

*“Everyone has the right to have access to health care services (…) and social security, including, if they are unable to support themselves and their dependants, appropriate social assistance.” (South Africa’s Bill of Rights)* [1]*.*

This quote highlights the progressive constitutional framework governing the country and emphasises post-apartheid aspirations for a healthy and economically-secure life for all people in the country. Major restructuring processes and expansion of both public health care and social security services have led to substantial progress in implementing these fundamental rights. While the DG has clearly played and continues to play an important role in averting some of the costs associated with disability arising from HIV/AIDS and treatment costs, the appropriateness of the grant is questionable for people living with HIV/AIDS. The introduction of a Chronic Illness Grant appears to be an appropriate alternative which is likely to encourage treatment adherence and avert the costs associated with the disease. In addition, it is imperative for collaborative efforts between the Departments of Social Development and Health to streamline and reduce the complexity of the DG application process and inform and prepare health care providers who are at the interface between social security and potential recipients. In the absence of such efforts, poor knowledge of the grants process and eligibility criteria on the part of patients and health care providers will continue to lead to poor treatment access.

## Additional files

Additional file 1: **ART patient exit interview.**

Additional file 2: **ART patient interview guide.**

## Abbreviations

SA: South Africa
ART: Antiretroviral treatment
DG: Disability Grant
SASSA: South African Social Security Agency
AOR: Adjusted odds ratio
TB: Tuberculosis

## Footnotes

1 The Social Assistance Act (No. 13 of 2004) defines a disable person as an adult who is ‘owing to his or her physical or mental disability, unfit to obtain, by virtue of any service employment or profession, the means needed to enable him or her to provide for his or her maintenance’.

2 Overall, there are currently seven types of social grants including not only the Disability Grant but also Older Persons Grant, Child Support Grant, Care Dependency Grant, Foster Child Grant, War Veterans Grant and the Grant-in-Aid (Department of Social Development in South Africa 2010).

3 People living with HIV/AIDS qualify for and receive a temporary DG, based on an assessment of the disability by a state-doctor. The assessment of disability rests with the doctor who is usually guided by the person’s CD4 count (less than 200) and/or TB/pneumonia related illness. People with a CD4 count of below 200 will usually qualify for the DG. At the same time, some people with CD4 counts of less than 200 may still be assessed as fit enough to work. Equally some people with CD4 counts above 200 who are very sick with TB or may qualify for the grant.

4 If the person’s CD4 count increases above 200 with ART and he/she is judged as fit for work by the doctor, they will no longer qualify for the DG.

5 The study was conducted between 2008 and 2010

6 In this study, expenditure is used as proxy for household income
