# Supplementary material for: Disability Grant: a precarious lifeline for HIV/AIDS patients in South Africa
Source: BMC Health Serv Res. 2015 Jun 9;15:227. doi: 10.1186/s12913-015-0870-8 (PMC4459666; doi:10.1186/s12913-015-0870-8)
Supplement: Supplementary file 2 — ART patient interview guide. [file 12913_2015_870_MOESM2_ESM.docx]

**REACH Patient Interview Guide**

**Antiretroviral Therapy**

**Before we start each interview:**

Begin with an opening statement to introduce the interviewer, the topic and purpose of the interview and the research. Outline interviewees’ rights (right not to answer specific questions, withdraw at any point, confidentiality, further questions at this stage?). Get the participant to sign the consent forms.

**Moving to the interview:**

1) Before we start talking about living with HIV, I would like to get to know a bit about you. Can you tell me about the major events in your life? Perhaps we can start with when and where you were born?

**Introduce the timeline.**

2) Let’s now talk about living with HIV. When did it start and what happened?

[prompts: What happened next? And then? Tell me more about…].

**Timeline cont.**

2a) How are you coping with living with HIV?

*3a)* Is this the first time that you have been sick in this way? [If not], please will you tell me about the other time(s) you had these symptoms. When did it start and what happened?

3b) How long have you been unwell/were you unwell for? When did you first get sick and what happened?

**Timeline cont.**

For each care-seeking event:

4) Can you describe a ‘typical’ visit (to the clinic/TH etc), including your journey of getting there and back?

5a) Can you tell me about a visit that stands out for you/that you remember?

5b) Can you tell me about a time that you had a good visit? What made it good?

5c) Can you tell me about a time that you had a visit that was less good? What made it so?

6) In accessing this treatment, do you feel that you were/are treated fairly? Please tell me more.

7a) Are you currently attending a clinic/hospital for any other services or treatments?

If so

7b) Are you able to get everything you need at the same clinic/during the same visit? Please can you tell me more?

8) How is your life now different to how it was before you got ill?

9) Do you think your illness has changed how other people behave towards you? If so, please can you tell me more about these changes?

10) How has your (current) treatment changed your life?

11) Do you think that getting treatment has changed the way that other people behave towards you? If so, please can you tell me more about these changes?

12) Is there anything you would like to add, which can help us to understand your experience?

13) Before we say good bye, please can I get/confirm some basic details from you, like your:

- age,
- home language,
- employment status,
- marital status, and
- who else lives in your household.

If these details have already emerged through the story, ‘answer’ them to show that you have listened to the respondent.

14) Have you got any further questions? If there are, provide answers to those and offer information contacts/ leaflets.

Thank participant for their time, input and willingness to talk. Ask if they would be willing for us to contact them again. If so, what would be the best way to agree on a meeting (get cell number). If they are willing to be contacted again, explain that we may follow up with them in a few weeks if we have any further questions. Leave an updated referral list with relevant service provider contact details, including counselling services.
